# Specialist care at a distance: Patient-reported experience with telemedicine video consultations for neurological symptoms associated with post-COVID-19 condition or in temporal association with COVID-19 vaccination in a single-center retrospective cross-sectional study

**DOI:** 10.1177/20552076261451372

**Published:** 2026-06-08

**Authors:** Vanessa Raeder, Fabian Boesl, Benno Bremer, Ameli Breuer, Heinrich J. Audebert, Christiana Franke

**Affiliations:** 1 Charité – Universitätsmedizin Berlin, corporate member of Freie Universität Berlin and Humboldt Universität zu Berlin, Department of Neurology and Experimental Neurology, Hindenburgdamm, Berlin, Germany

**Keywords:** telemedicine, video conferencing, patient satisfaction, post-COVID-19 condition, vaccination, cognitive dysfunction, fatigue

## Abstract

**Objectives:**

To describe patient-reported experience with a structured telemedicine video consultation (TVC) pathway in routine neurological care among adults with persistent neurological symptoms with onset after SARS-CoV-2 infection or in temporal association with COVID-19 vaccination.

**Methods:**

This single-center retrospective cross-sectional study examined TVC delivered in routine clinical care at the Department of Neurology, Charité-Universitätsmedizin Berlin (11/2022–12/2023). Patients with neurological symptoms persisting ≥3 months after SARS-CoV-2 infection or occurring in temporal association with COVID-19 vaccination were included. Data included demographics, symptom assessments, validated questionnaires on mood, fatigue, and subjective memory, audiovisual cognitive screening, and a post-consultation survey of patient-reported telemedicine experience.

**Results:**

Forty-one patients (mean age 41.3±12.8 years; 61% female) were included. The median home-to-clinic distance was 356 km; and 39% reported mobility limitations affecting in-person attendance. Patients reported positive immediate experiences: most found scheduling easy, reported good audio/video quality, and considered the consultation relevant and time-saving. A minority reported technical difficulties or required assistance. Patients reported high fatigue levels (Fatigue Severity Scale 5.3±1.8), frequent subjective cognitive symptoms, moderate subjective memory satisfaction (Multifactorial Memory Questionnaire-Satisfaction Domain, 47.7±17.3), and mean Montreal Cognitive Assessment score of 27.3±2.3.

**Discussion:**

These exploratory findings indicate positive patient-reported experience with a structured TVC pathway in routine neurological care. Fatigue and subjective cognitive difficulties were common across the selected cohort. This study describes a tele-neurology workflow integrating document pre-review, digital symptom assessment, remote history taking, and cognitive screening. Further controlled longitudinal studies are needed to determine the clinical role, safety, and equitable implementation of such approaches.

## 1. Introduction

Neurological telemedicine has advanced significantly over the past twenty years, with the Coronavirus Disease 2019 (COVID-19) pandemic accelerating adoption of remote patient care.^1–4^

Post-COVID-19 condition (PCC) occurs in a proportion of individuals after SARS-CoV-2 infection, with global prevalence estimates ranging from 2.5% to 63.9%, potentially reaching 37.6% among hospitalized patients.^5,6^ PCC frequently involves debilitating neurological symptoms like severe fatigue and cognitive deficits,^7,8^ impairing daily life and impeding access to care, as well as a potential social and personal burden adversely affecting global public health.^9,10^ Similar challenges arise in a subset of patients reporting new-onset symptoms following COVID-19 vaccination. While these clinical presentations are frequently referred to as “post-COVID-19 vaccine syndrome” (PVS) in case series,^11,12^ it is important to emphasize that unlike PCC, PVS currently lacks a globally accepted case definition, WHO-validated criteria, or ICD code classification. Therefore, in the context of this study, PVS is used as a descriptive label for neurological symptoms occurring in temporal association with vaccination, rather than a confirmed causal diagnosis. Currently, diagnostic certainty for PVS remains limited, and both conditions lack definitive biomarkers and treatments, posing diagnostic difficulties.^13^ Despite the ongoing debate regarding the diagnostic status of PVS, patients attributing their symptoms to vaccination present with substantial suffering and healthcare needs. As these patients presented to the same outpatient clinic, they were included for descriptive characterization alongside the PCC cohort. Access to specialized care often requires travel, which may represent an important barrier for some patients, particularly those living in rural areas or those with severe symptoms.

Telehealth services delivered via videoconferencing can approximate selected aspects of in-person consultations and have been used across a range of age groups and health conditions.^14^ During the COVID-19 pandemic, telemedicine became essential for maintaining healthcare continuity while minimizing in-person contact.^15^ Telemedicine has expanded from its initial focus on stroke to broader neurological applications and beyond.^1,4^ This development has been facilitated by the removal of previous barriers such as credentialing restrictions.^16^

However, data remain limited on how patients with neurological symptoms associated with PCC or in temporal association with COVID-19 vaccination experience telemedicine video consultation (TVC) in routine clinical care. The aim of this study was to describe patient-reported experience with TVC in routine neurological care and to provide a descriptive characterization of patients presenting with neurological symptoms following SARS-CoV-2 infection or those with self-reported symptoms temporally associated with COVID-19 vaccination. The study was not designed to assess clinical effectiveness, safety, or equivalence to in-person care.

## 2. Material and methods

This single-center, retrospective cross-sectional study described patient-reported experience with TVC delivered in a real-world routine neurological care setting. The TVC outpatient clinic was specifically established for patients with neurological symptom onset in temporal association with either COVID-19 disease (PwPCC) or COVID-19 vaccination (hereinafter referred to as PwPV to describe the cohort, explicitly acknowledging the lack of established diagnostic criteria and biomarkers for this group) at the Department of Neurology at the Charité, Berlin, Germany.

The Institutional Review Board of the Charité approved the study (Institutional Review Board number EA2/102/22). Written informed consent was waived by the Institutional Review Board due to the observational and retrospective nature of the study and in accordance with laws and regulations of the Federal State of Berlin that allows the scientific use of pseudonymized data collected within routine-care documentation and quality monitoring of individual hospitals (§25 Berliner Landeskrankenhausgesetz).^17^

**Figure 1.**
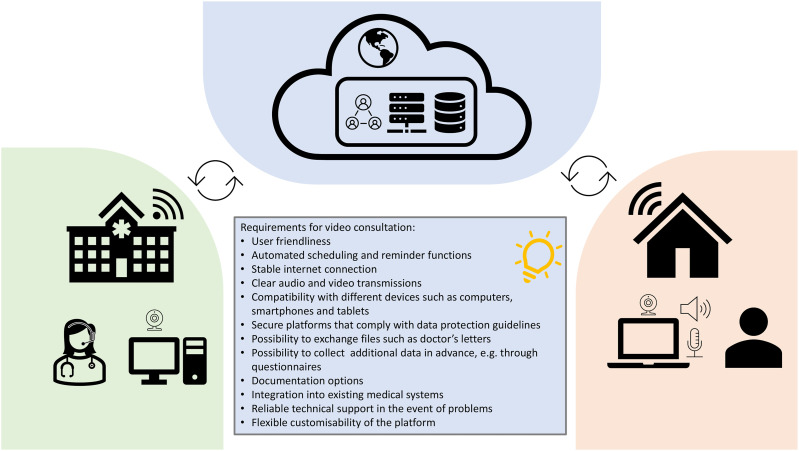
Requirements for video consultations.

Patients were enrolled in a one-hour outpatient TVC conducted as part of routine care and were invited to complete a post-consultation survey assessing patient-reported experience with the telemedicine consultation, including administrative, technical, and perceived consultation-related aspects. The aim of the TVC was to provide guidance and support on further diagnostic and therapeutic options for these patients while advising them about possible opportunities in observational or treatment studies. Depending on their condition, the TVC were carried out either individually with the patient or with the patient’s caregivers. Inclusion criteria for this consultation were adult patients (at least 18 years of age) with documented SARS-CoV-2 infection by PCR or a COVID-19 vaccination record, with new-onset neurological symptoms such as persistent memory, attention and concentration difficulties, and fatigue within 12 weeks of documented acute SARS-CoV-2 infection or persistent symptoms occurring in temporal association with COVID-19 vaccination. Patients were able to independently inform themselves about the consultation criteria via the Charité website and book appointments directly on the designated online platform, without needing prior contact with the clinic. The TVC was designed for patients presenting with neurological symptoms that had newly emerged following a COVID-19 disease or in temporal association with COVID-19 vaccination. The service was therefore primarily intended for patients without known pre-existing neurological conditions prior to SARS-CoV-2 infection/vaccination, in order to focus on post-infectious or post-vaccination neurological manifestations. Severe neurological and psychiatric conditions were defined as exclusion criteria for the post-consultation survey. These TVC were provided as a service for which patients incurred a consultation fee. This service was offered in parallel with in-person consultations on site and was conducted by a neurologist via TVC. Figure 1 illustrates technical and functional considerations relevant to conducting TVC in this clinical context.

Prior to the consultations, demographic data and symptom assessments were collected via the telemedicine platform.

Additionally, established questionnaires to assess depression, fatigue, and memory satisfaction were digitally collected using the platform’s online forms, which included the Beck Depression Inventory II (BDI-II),^18^ the Fatigue Severity Scale (FSS),^19^ and the Memory Satisfaction sub-domain of the Multifactorial Memory Questionnaire (MMQ).^20^ Patients could upload their previous medical records to the platform, which were then thoroughly reviewed by the clinician prior to the appointment. Patients were proactively reminded to complete their data by the clinician via the platform if they had not completed the questionnaires or uploaded medical records prior to the appointment. This approach was intended to ensure that all relevant information was available for review, aiming to facilitate a more informed and efficient consultation process. Patients were also encouraged to ask any important questions prior to their appointment through the platform chat or email. All patients automatically received reminders of their upcoming telemedicine appointments and instructions on starting the TVC by email and via the online platform.

During the TVC, a detailed medical history was taken to record current complaints. Cognitive symptoms were specifically assessed using the Montreal Cognitive Assessment (MoCA) Audiovisual as a screening tool.^21^ If necessary, a brief physical examination was conducted. Based on the results of the initial consultation, patients who required further evaluation were either scheduled for an in-person post-COVID-19 neurological consultation at the study clinic or referred to a local facility, depending on their place of residence, for additional diagnostics and treatment adjustments as needed. This strategy offered symptomatic guidance and helped identify cases in which in-person assessment or local referral appeared clinically warranted.

At the end of the consultation, when the patients had no further questions, they were asked whether they would like to complete a quality management questionnaire to evaluate their experience with telemedicine. Patient-reported experience was assessed using a structured questionnaire developed for internal quality management. This questionnaire comprised dichotomous (yes/no) items across three key domains: accessibility (ease of scheduling, clarity of instructions), technical fidelity (audio/video quality, stability), and consultation-related perceptions (comparison to in-person care, time efficiency, adequacy of duration). Additional items evaluated patient mobility and service recommendation, alongside multiple-choice items regarding device and network usage. The questionnaire was not psychometrically validated or designed to assess objective clinical quality, safety, or effectiveness. All patients were given the opportunity to verbally explain their answers, which were recorded as free-text comments for descriptive consideration; no formal qualitative analysis was performed.

### 2.1. Statistical analysis

Statistical analysis was performed using Microsoft Excel and IBM SPSS 26. Descriptive statistics were used to summarize demographic and clinical data. Due to the unequal sample sizes and non-normal distribution of the data, group comparisons were conducted using the non-parametric Mann-Whitney U test for continuous variables and the Chi-square test for categorical variables. Given the exploratory nature and small sample size, all comparisons are descriptive and should not be interpreted as confirmatory hypothesis testing. No correction for multiple testing was applied and no effect sizes were calculated; therefore, p-values are provided only as descriptive indicators and should not be used to infer equivalence or diagnostic group differences.

## 3. Results

Between November 2022 and December 2023, 42 patients with neurological symptoms participated in a TVC with a neurologist. Of these, 26 were classified as PwPCC and 15 as PwPV, based on the temporal association of their neurological symptoms with either acute SARS-CoV-2 infection or COVID-19 vaccination. One patient had to be excluded due to severe psychiatric symptoms requiring inpatient admission.

The demographic profile was comparable between the two groups in terms of gender (p=0.558) and age (p=0.198). On average, PwPCC were slightly younger (39.0 years) than PwPV (45.3 years) (Table 1). The PwPCC also showed descriptively higher employment rates and higher sick leave than PwPV. Further patient characteristics, including detailed alcohol consumption and smoking status, are also summarized in Table 1.

**Table 1.** Sociodemographic characteristics of the study population.

Characteristic	PwPCC (n=26)	PwPV (n=15)	Total (n=41)
Age (years)			
Mean ± SD	39.0 ± 11.9	45.3 ± 13.3	41.3 ± 12.8
Range (Min - Max)	19 - 59	30 - 71	19 - 71
**Sex, n (%)**			
Female	17 (65.4)	8 (53.3)	25 (61.0)
Male	9 (34.6)	7 (46.7)	16 (39.0)
**Employment Status, n (%)**			
Employed - working	8 (30.8)	7 (46.7)	15 (36.6)
Employed - on medical leave	15 (57.7)	5 (33.3)	20 (48.8)
Actively seeking a job	1 (3.8)	0 (0.0)	1 (2.4)
Not seeking a job	2 (7.7)	3 (20.0)	5 (12.2)
**Smoking Status, n (%)**			
Never smoked	21 (80.8)	13 (86.7)	34 (82.9)
Former smoker	3 (11.5)	1 (6.7)	4 (9.8)
Current smoker	2 (7.7)	1 (6.7)	3 (7.3)
**Alcohol Consumption, n (%)**			
History of alcohol abuse	0 (0.0)	1 (6.7)	1 (2.4)
Current abstinence	26 (100.0)	15 (100.0)	41 (100.0)
**Distance to clinic (km)**			
Median (IQR)	352 (182.3, 490.0)	356 (146.5, 553.0)	356 (151.0, 532.0)
Range (Min - Max)	9 - 2300	2 - 709	2 - 2300
**Pre-existing Comorbidities found with detailed analysis of medical records**			
Neurological disorder	6 (23.1)	4 (26.7)	10 (24.4)
Psychiatric disorder	6 (23.1)	1 (6.7)	7 (17.1)
**Consultation & Participation**			
Assistance from caregiver/family during medical history	7 (26.9)	1 (6.7)	8 (19.5)
Unable to participate	1 (3.8)^a^	0 (0.0)	1 (2.4)

Data are presented as mean ± standard deviation (SD), median (Interquartile Range; IQR), or count (percentage; %).

*Abbreviations:* PwPCC: patients with neurological symptom onset in temporal association with COVID-19 disease; PwPV: patients with neurological symptom onset in temporal association with COVID-19 vaccination.

^a^One PwPCC (3.8%) was unable to complete the video consultation due to severe fatigue.

**Figure 2.**
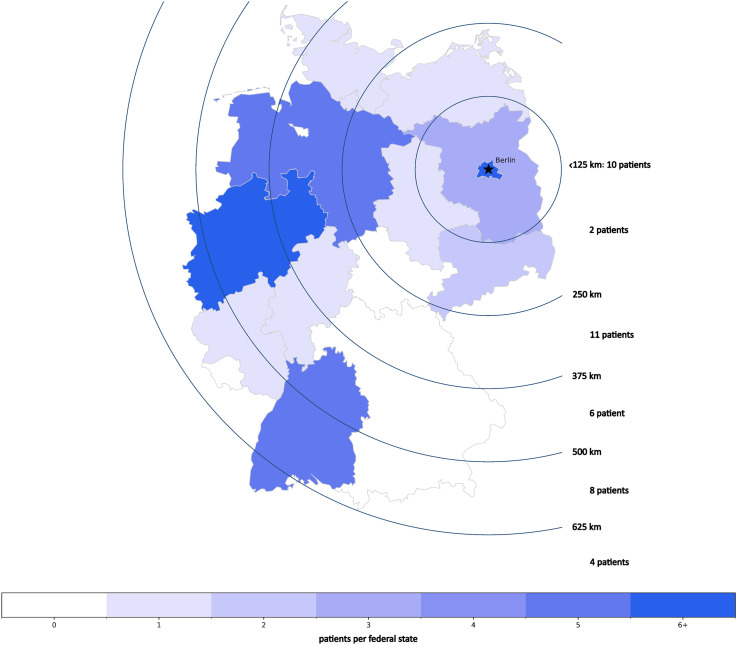
Geographical distribution of all patients per German federal state (map of Germany) and by radius between 125 km (77.7 miles) distances (blue radii).

The median distance to the clinic was 356 km (221 miles) and was nearly identical for both groups: 352 km (218 miles) for PwPCC and 356 km (221 miles) for PwPV. Distance varied widely among participants (Figure 2; Table 1). The minimum distance to the clinic was 2 km (1.2 miles), while the maximum distance was 2,300 km (1,429 miles), indicating that the service was used by patients living at varying distances from the clinic. Only 9 patients (22%) resided within the local metropolitan catchment area (<50 km), while the majority resided outside this local catchment area.

Despite the guidance provided on the telemedicine platform, several patients with neurological or psychiatric conditions proceeded to make bookings (Table 1). Three patients were hospitalized due to the onset of acute symptoms. None of the patients required intensive care treatment, with or without intubation or additional respiratory support such as CPAP or supplemental oxygen.

Within the PwPCC cohort, the earliest reported positive SARS-CoV-2 PCR test dated back to February 2020, and the most recent was reported in November 2022. The median date of the first positive test was in March 2022. All reported cases were classified as mild, with patients experiencing up to three successive SARS-CoV-2 infections.

COVID-19 vaccination patterns (Supplement 1) among both patient groups were analyzed based on vaccine type (BNT162b2, mRNA-1273, and ChAdOx1-S) and dose number.^22–24^ BNT162b2 was the most frequently administered initial COVID-19 vaccine in both groups. In the PwPV, symptom onset was most frequently reported after the first dose (47%), followed by the second and third doses (20% each). No clear temporal association between vaccination and symptom onset was observed in the PwPCC.

Table 2 illustrates the extensive diagnostic procedures patients underwent before the TVC. One patient was diagnosed with small fiber neuropathy of unknown cause, confirmed by skin punch biopsy revealing reduced intraepidermal nerve fiber density, with a presumed link to infection-triggered immune dysregulation following COVID-19. Four PwPCC had previously undergone therapeutic plasma exchange, and one PwPV had received immunomodulatory treatment with methotrexate and prednisolone. Two PwPCC had already undergone rehabilitation treatment due to post-COVID-19 symptoms.

**Table 2.** A summary of previous diagnostic procedures, as documented in the medical records.

Diagnostic procedures	PwPCC (n=26)		PwPV (n=15)		Total (n=41)	
	n	%	n	%	n	%
Cranial Magnetic Resonance Imaging	21	80.8%	13	86.7%	34	82.9%
Cervical Spine Magnetic Resonance Imaging	6	23.1%	8	53.3%	14	34.1%
Whole-body Magnetic Resonance Imaging	1	3.8%	0	0.0%	1	2.4%
Cranial Computed Tomography	3	11.5%	3	20.0%	6	14.6%
Electroencephalography	9	34.6%	7	46.7%	16	39.0%
Electromyography	4	15.4%	2	13.3%	6	14.6%
Nerve Conduction Velocity	9	34.6%	5	33.3%	14	34.1%
Somatosensory Evoked Potentials	5	19.2%	1	6.7%	6	14.6%
Motor Evoked Potentials	3	11.5%	0	0.0%	3	7.3%
Visual Evoked Potentials	0	0.0%	1	6.7%	1	2.4%
Electrocardiogram	21	80.8%	12	80.0%	33	80.5%
Long-term Electrocardiogram	8	30.8%	5	33.3%	13	31.7%
Exercise Stress Test	0	0.0%	2	13.3%	2	4.9%
Transthoracic Echocardiography	17	65.4%	10	66.7%	27	65.9%
Cardiac Magnetic Resonance Imaging	4	15.4%	2	13.3%	6	14.6%
Pulmonary Function Test	15	57.7%	4	26.7%	19	46.3%
Chest X-ray	6	23.1%	3	20.0%	9	22.0%
Chest Computed Tomography	4	15.4%	1	6.7%	5	12.2%
Lumbar Puncture	7	26.9%	3	20.0%	10	24.4%
Physical Examination	18	69.2%	12	80.0%	30	73.2%
Punch Biopsy	2	7.7%	2	13.3%	4	9.8%
Extracranial Doppler Sonography	4	15.4%	6	40.0%	10	24.4%
Laboratory Tests	23	88.5%	15	100.0%	38	92.7%
**Total**	**190**	**31.8%**	**117**	**33.9%**	**307**	**32.6%**

*Abbreviations:* PwPCC: patients with neurological symptom onset in temporal association with COVID-19 disease; PwPV: patients with neurological symptom onset in temporal association with COVID-19 vaccination.

Self-reported symptom questionnaires were completed on the telehealth platform prior to scheduling a TVC, covering both acute symptoms during acute infection and persisting symptoms since infection or vaccination (Table 3; Table 4). In this cohort, fatigue was reported more frequently by PwPCC than by PwPV as a symptom experienced since infection or vaccination and PwPCC recorded higher fatigue level at time of TVC (Table 4; Table 5). In addition, PwPCC more often presented with cognitive deficits and sleep disturbances at the time of consultation. Overall, self-reported symptoms remained largely stable over time, with the exception of a decrease in headache prevalence among PwPCC. In PwPV, the most frequently reported symptoms were headaches or other pain.

**Table 3.** Self-reported symptoms during acute COVID-19 of patients presenting with post-COVID-19 condition (n=26).

Symptoms	*Yes*		*No*		*N/A*	
	*n*	*%*	*n*	*%*	*n*	*%*
Cold symptoms	22	84.6%	2	7.7%	2	7.7%
Dyspnea	14	53.8%	10	38.5%	2	7.7%
Fever	16	61.5%	8	30.8%	2	7.7%
Headache	22	84.6%	2	7.7%	2	7.7%
Limb pain	19	73.1%	5	19.2%	2	7.7%
Myalgia	14	53.8%	10	38.5%	2	7.7%
Dysosmia	9	34.6%	15	57.7%	2	7.7%
Dysgeusia	8	30.8%	16	61.5%	2	7.7%
Chills	11	42.3%	13	50.0%	2	7.7%
Abdominal pain	4	15.4%	20	76.9%	2	7.7%
Diarrhea	3	11.5%	21	80.8%	2	7.7%
Dizziness	12	46.2%	12	46.2%	2	7.7%

**Table 4.** Self-reported symptoms since acute COVID-19 (n=24) or COVID-19 vaccination (n=15) and current symptoms assessed during consultation of post-COVID-19 condition patients (n=25) and post-Vaccine patients (n=15).

Self-reported symptoms since acute COVID-19 or COVID-19 vaccination	PwPCC (n=24)		PwPV (n=15)		Total (n=39)	
	n	%	n	%	n	%
Concentration disturbances	23	95.8%	12	80.0%	35	89.7%
Attention disorders	16	66.7%	7	46.7%	23	59.0%
Memory disturbances	15	62.5%	8	53.3%	23	59.0%
Word finding difficulties	13	54.2%	7	46.7%	20	51.3%
Fatigue	21	87.5%	9	60.0%	30	76.9%
Headache	18	75.0%	10	66.7%	28	71.8%
Myalgia	18	75.0%	9	60.0%	27	69.2%
Muscle weakness	15	62.5%	7	46.7%	22	56.4%
Balance disorders	6	25.0%	7	46.7%	13	33.3%
Sensory disturbances	9	37.5%	7	46.7%	16	41.0%
Dizziness	15	62.5%	10	66.7%	25	64.1%
Dyssomnia	1	4.2%	0	0.0%	1	2.6%
Dysgeusia	0	0.0%	0	0.0%	0	0.0%
Dyspnea	8	33.3%	5	33.3%	13	33.3%
Tachycardia	10	41.7%	8	53.3%	18	46.2%
Chest pain	9	37.5%	4	26.7%	13	33.3%
Joint pain	11	45.8%	3	20.0%	14	35.9%
Gastrointestinal symptoms	10	41.7%	5	33.3%	15	38.5%
Intermittent fever	3	12.5%	0	0.0%	3	7.7%
Sleep disorders	14	58.3%	6	40.0%	20	51.3%
Anxiety	5	20.8%	6	40.0%	11	28.2%
Depression	4	16.7%	1	6.7%	5	12.8%
Irritability	6	25.0%	5	33.3%	11	28.2%
**Current Symptoms assessed during consultation**	**PwPCC (n=25)**		**PwPV (n=15)**		**Total (n=40)**	
	**n**	**%**	**n**	**%**	**n**	**%**
Cognitive deficits	19	76.0%	6	40.0%	25	62.5%
Fatigue	22	88.0%	8	53.3%	30	75.0%
Sleep disorders	13	52.0%	2	13.3%	15	37.5%
Other Pain	13	52.0%	9	60.0%	22	55.0%
Headache	15	60.0%	9	60.0%	24	60.0%
Tinnitus	5	20.0%	0	0.0%	5	12.5%
Sight disorder	4	16.0%	4	26.7%	8	20.0%
Brain fog	3	12.0%	1	6.7%	4	10.0%
Sensory overload	8	32.0%	5	33.3%	13	32.5%
Anxiety	3	12.0%	3	20.0%	6	15.0%
Paresthesia	8	32.0%	3	20.0%	11	27.5%
Hypesthesia	3	12.0%	5	33.3%	8	20.0%
Pallhypesthesia	3	12.0%	0	0.0%	3	7.5%
Myasthenia	6	24.0%	5	33.3%	11	27.5%
Palpitations	6	24.0%	4	26.7%	10	25.0%
Additional symptoms	19	76.0%	14	93.3%	35	87.5%

*Abbreviations:* PwPCC: patients with neurological symptom onset in temporal association with COVID-19 disease; PwPV: patients with neurological symptom onset in temporal association with COVID-19 vaccination.

**Table 5.** Comparison of clinical questionnaire scores between groups.

Questionnaire & Statistic	PwPCC (n=26)	PwPV (n=15)	Total (n=41)	P-value*
Montreal Cognitive Assessment (MoCA)				
n (completed)	18	5	23	0.597
Score, Mean ± SD	27.4 ± 2.4	26.8 ± 2.3	27.3 ± 2.3	
Score Range (Min - Max)	23 - 30	24 - 30	23 - 30	
**Beck Depression Inventory II (BDI-II)**				
n (completed)	26	15	41	0.385
Score, Mean ± SD	13.2 ± 7.1	11.3 ± 5.6	12.5 ± 6.5	
Score Range (Min - Max)	3 - 31	5 - 27	3 - 31	
**Fatigue Severity Scale (FSS)**				
n (completed)	26	15	41	**<0.05**
Score, Mean ± SD	5.8 ± 1.4	4.3 ± 1.9	5.3 ± 1.8	
Score Range (Min - Max)	1.5 - 7.0	1.7 - 7.0	1.5 - 7.0	
**Multifactorial Memory Questionnaire - Satisfaction Domain (MMQ)**				
n (completed)	24	14	38	**<0.05**
Score, Mean ± SD	43.0 ± 16.7	55.6 ± 15.9	47.7 ± 17.3	
Score Range (Min - Max)	11 - 72	16 - 72	11 - 72	

Data are presented as mean ± standard deviation (SD).

*P-values were calculated using Mann-Whitney U test to compare PwPCC and PwPV groups. However, due to the small sample size and observational design, these statistical comparisons are exploratory in nature. They should be interpreted with caution and are not intended to confirm diagnostic differences or clinical efficacy.

*Abbreviations:* PwPCC: patients with neurological symptom onset in temporal association with COVID-19 disease; PwPV: patients with neurological symptom onset in temporal association with COVID-19 vaccination.

Self-reported symptoms of depression, as measured by the BDI-II, were similar between the groups (Table 5). The FSS revealed higher fatigue levels in PwPCC compared to PwPV. Self-reported memory function, as assessed by the MMQ, indicated lower scores in PwPCC, reflecting lower subjective memory satisfaction. Objective cognitive performance, as reflected by MoCA scores, was comparable between the two groups.

A brief physical examination via TVC identified one PwPCC presenting with a right-sided monoparesis of unclear etiology, as well as PwPV and PwPCC exhibiting signs indicative of functional gait disorder, impaired balance, and gait instability. However, definitive clinical assessments were not achievable in all cases due to inherent diagnostic limitations of TVC. Affected patients were subsequently referred for an in-person evaluation or inpatient care.

Overall, patient-reported responses to the post-consultation experience questionnaire indicated high immediate acceptance and satisfaction with TVC in this selected cohort (Table 6). Over 95% reported easy scheduling, clear instructions, good audio/video quality, and appropriate consultation duration. 97.6% of patients also agreed with the questionnaire item “just as effective as an in-person consultation”; which reflects immediate subjective perception and does not constitute evidence of objective equivalence in diagnostic accuracy, clinical effectiveness, or safety. All participants found TVC to be relevant and time-saving and indicated that they would recommend it to others.

**Table 6.** Patient-reported experience questionnaire on telemedicine video consultations (N = 41).

Questions	*Yes*		*No*		*N/A*	
	*n*	*%*	*n*	*%*	*n*	*%*
Was easy to schedule	39	95.1%	2	4.9%	0	0.0%
Had clear instructions	41	100.0%	0	0.0%	0	0.0%
Was the audio/video quality good	40	97.6%	1	2.4%	0	0.0%
Had technical support by another person	8	19.5%	33	80.5%	0	0.0%
Had technical issues	5	12.2%	36	87.8%	0	0.0%
Was just as effective as in-person consultation	40	97.6%	0	0.0%	1	2.4%
Would suggest improvements	3	7.3%	38	92.7%	0	0.0%
Would be mobile enough to visit in person	25	61.0%	16	39.0%	0	0.0%
Would schedule in-person consultation if video consultation not possible	29	70.7%	12	29.3%	0	0.0%
Duration of the video consultation was adequate	40	97.6%	1	2.4%	0	0.0%
Does save time	41	100.0%	0	0.0%	0	0.0%
Regards video consultation as relevant	41	100.0%	0	0.0%	0	0.0%
Would recommend to others	41	100.0%	0	0.0%	0	0.0%
**Which device was used?**	​	​	​	​	​	​
*Android – Smartphone*	3	7.3%	​	​	​	​
*Android – Tablet*	3	7.3%	​	​	​	​
*iPad - Tablet*	8	19.5%	​	​	​	​
*iPhone – Smartphone*	2	4.9%	​	​	​	​
*Laptop*	21	51.2%	​	​	​	​
*PC with camera and microphone*	4	9.8%	​	​	​	​
**Internet connection**	​	​	​	​	​	​
*Cellular network*	1	2.4%	​	​	​	​
*Fixed network*	40	97.6%	​	​	​	​

Laptops were the most commonly used device (51%), typically connected via fixed networks (98%) (Table 6). A few patients experienced technical issues (12%) or required third-person technical support (20%). The latter subgroup differed from the overall cohort in several baseline characteristics: they were older (mean age 49.6 vs. 41.3 years), showed lower cognitive performance (MoCA 25.8 vs. 27.3), reported more depressive symptoms (BDI-II 15.5 vs. 12.5), and rated their memory poorer (MMQ 42.4 vs. 47.7).

Medical reports were uploaded directly through the portal by 65.9% of patients, while the remainder used alternative on- or off-platform channels. To encourage pre-visit uploads, 43.9% of patients received up to three reminder emails.

Notably, 39% of patients reported being unable to attend in-person. More PwPCC (27%) than PwPV (7%) were supported by caregivers or family members during the assessment of medical history. One PwPCC (4%) was physically unable to take part in the TVC due to her severe fatigue.

## 4. Discussion

The present study describes patient-reported experience with TVC delivered as part of a real-world routine clinical care setting in a specialized neurology outpatient clinic for PwPCC and PwPV.

The demographic profiles appeared broadly similar between the groups, with a predominance of younger female participants, consistent with prior study reports involving PwPCC and PwPV.^11,25^ Given the small and numerically unbalanced sample, however, subgroup comparisons are descriptive and exploratory only. Demographic data further showed that the service was used by patients living both near and far from the clinic. The median patient-reported distance of 356.0 km (221.2 miles) indicates that some patients used the service across considerable geographic distances, although this finding alone does not establish improved access compared with standard in-person care. This observation is in line with prior telemedicine literature suggesting that remote consultations can reduce travel requirements in some settings; previous studies have reported average travel savings per consultation of 447.4 km (278 miles), with associated reductions in travel costs and environmental burden.^26^

At the same time, related telemedicine literature suggests that uptake may be shaped by socioeconomic and demographic inequities.^27,28^ Digital literacy and access to reliable internet or mobile data may further limit equitable use of remote care.^29^ Access to the present service was shaped by self-referral via an online booking platform, digital literacy, stable internet access, and the ability to pay the consultation fee. These factors likely selected for patients who were already able and willing to engage with telemedicine and may have limited participation by more disadvantaged individuals. In addition, 39% of patients reported limited mobility or incapacitating symptoms, most notably severe fatigue. For such patients, travel to specialist care may itself represent a barrier, particularly given concerns about post-exertional symptom exacerbation.^30^ In this sense, a telemedical format may reduce travel-related burden for selected individuals; however, our study design does not allow conclusions about broader access effects at the population level. This cautious interpretation is consistent with prior work indicating that, even in high-income settings, access to specialist care may be restricted by distance and disability.^31^

Regarding the clinical presentation, patients in both groups frequently reported perceived fatigue and cognitive difficulties. Headaches and other pain symptoms appeared more prominent in reports from PwPV during the consultation. Fatigue and cognitive impairments are commonly reported neurological symptoms in both PCC and patients with symptoms temporally associated with COVID-19 vaccination.^11,32,33^ Pain, including headache and neuropathic pain, is also frequently described.

Symptom assessment during the TVC was informed by pre-consultation questionnaires, booking information, verbal history taking, and uploaded documents. This multimodal information may have helped structure the clinical assessment during the TVC.

Validated self-report questionnaires (BDI-II, FSS, MMQ) were integrated into routine telemedicine assessments and supported the structured documentation of symptom burden and perceived cognitive difficulties. Overall, patient-reported fatigue and perceived cognitive difficulties were prominent and broadly consistent with previous literature.^11,34^ These findings suggest that such measures may support structured symptom assessment and documentation, and inform clinical interpretation in telemedical consultations.

The MoCA provided a brief measure of global cognition in patients reporting cognitive symptoms and did not suggest marked impairment at the group level. This finding should be interpreted cautiously, however, as brief cognitive screening may not capture subtle or domain-specific deficits, and average scores may obscure heterogeneity within the sample. In addition, MoCA findings were not interpreted against age- and education-adjusted normative data. The high subjective burden reported in the symptom questionnaires may therefore reflect a discrepancy between perceived cognitive difficulties and screening-based global cognitive performance. In younger and high-functioning individuals, even subtle cognitive changes may be experienced as highly disruptive in everyday life and may contribute to a substantial subjective burden.^35^

Previous diagnostic work-up appeared heterogeneous across the cohort and included investigations such as cervical spine MRI and pulmonary function testing, likely reflecting the diversity of reported symptoms and referral pathways.^11,36^ Given the absence of established diagnostic criteria or ICD codes for PVS, and the lack of validated diagnostic parameters and specific biomarkers for PCC and PVS, compounded by complex symptom presentations and pre-existing conditions, it remains challenging to definitively rule out alternative etiologies for symptom classification in these contexts, underscoring the need for further research.^7,13^ In this setting, a structured pre-consultation review of patient-uploaded documents and prior findings may help organize the TVC and support a systematic consideration of relevant differential diagnoses, particularly in complex cases with comorbidities. Some patients also reported prior use of immunomodulatory treatment and apheresis, indicating that parts of this cohort had already engaged with experimental or off-label management approaches before the TVC. Such approaches have been discussed in the context of PCC and chronic fatigue syndrome, including in relation to proposed mechanisms such as immune dysregulation and autoantibody-related processes.^7,37,38^

Conducting a comprehensive neurological physical examination via TVC remains limited by technical constraints, variable patient digital literacy, and the lack of widely validated standardized methods for remote neurological examination. In particular, the absence of tactile examination limits the evaluation of muscle tone, reflexes, plantar responses, and subtle sensory deficits.^39^ Strength testing relies on indirect observation and technical conditions, introducing uncertainty and the potential for false-negative findings; coordination, gait, and cranial nerve assessment are likewise limited. These limitations necessitate a low threshold for recommending in-person evaluation when diagnostic uncertainty persists or a tactile neurological examination is required. TVC may therefore serve as a supportive component of specialist assessment rather than a substitute for an on-site neurological examination. In the present study, a cautious supportive diagnostic approach was adopted: in cases of diagnostic ambiguity, limited assessability in the TVC setting, including inconclusive virtual examination findings, or suspected pathology requiring tactile neurological examination, patients were advised to attend an in-person evaluation. Although randomized studies in other neurological subspecialties, such as multiple sclerosis and non-acute headache, have reported outcomes broadly comparable to in-person care under defined conditions,^40,41^ transferability to post-infectious or post-vaccination symptom presentations remains uncertain.

Participants reported predominantly positive immediate post-consultation experiences with the TVC. Within this framework of patient-reported experience, participants generally described the consultations as relevant to them and indicated that they would recommend the service to others. Scheduling was generally perceived as easy, instructions as clear, and audio and video quality as satisfactory. Reported time savings were mainly attributed to reduced travel requirements and shorter waiting times, in line with previously described benefits of telemedicine.^26,42^ At the same time, at least one patient explicitly preferred a traditional in-person consultation, citing a preference for face-to-face interaction and being physically present with the physician, thereby highlighting a known limitation of telemedicine.^14^ Overall, these findings indicate a high immediate acceptability of the TVC format in this selected cohort. However, this should not be interpreted as evidence of clinical adequacy or effectiveness, as these factors may have been influenced by self-selection, acquiescence or social desirability bias.^43,44^ Additionally, the fee-based nature of the service may have influenced patient expectations, perceived value, or satisfaction ratings, although the direction and magnitude of this effect remain uncertain.^45^ Moreover, the post-consultation ratings were based on a non-validated questionnaire with mainly dichotomous response options, which may have limited response discrimination and contributed to ceiling effects.^46^

Despite high immediate acceptability in this selected cohort, technology-specific barriers remain among the major challenges to telemedicine adoption worldwide.^47^ Beyond infrastructural issues such as unstable internet connections and limited availability of personal hardware reported in the literature,^48^ limited digital proficiency also appeared to represent a usability challenge in the present cohort. Patients requiring technical assistance appeared to be older and to have greater cognitive difficulties. This is consistent with previous findings that older adults and individuals with cognitive impairment are more likely to experience difficulties with telemedicine platforms.^49^ At the same time, these barriers may be underestimated in the present study, as participation itself required a minimum level of digital access and engagement.

However, technical readiness alone is insufficient. Institutional inertia, outdated policy frameworks, and persistent social inequities, particularly the digital divide, have repeatedly been identified as major determinants of whether telemedicine in neurology translates into broader access in practice.^31^ If left unaddressed, these barriers may substantially limit the access benefits telemedicine can provide.

The frequent involvement of family members or caregivers suggests that TVC platforms may need to better support the participation of multiple users, rather than relying on informal workarounds. In addition, TVC has often been associated with younger user groups.^50^ This may have contributed to the observed age distribution and may limit the generalizability to older or less digitally confident populations.

Although telemedical assessment remains dependent on technical stability, the patient’s ability to participate, and, in some cases, caregiver support, TVC may represent a supportive component of specialist neurological care for selected patients.^39^ Wearable devices and remote monitoring technologies are also being explored in current research.^51^ Further details on the benefits and challenges of TVC are provided in Supplement 2. Taken together, the value of this TVC model in this setting lay less in replacing in-person neurological assessment than in providing a structured initial specialist contact that combined pre-consultation document review, digital symptom assessment, focused remote history taking, brief cognitive screening, and referral for local or on-site follow-up when clinically indicated.

This study is subject to several limitations that constrain its interpretation. First, this was a small, single-center, retrospective cross-sectional study without longitudinal follow-up. The cohort size (N = 41) and unequal group distribution (PwPCC n = 26 vs. PwPV n = 15) may have affected the sensitivity and interpretability of comparative analyses. Non-parametric tests were used in light of these imbalances, but statistical power remained limited; therefore, any observed between-group differences should be considered descriptive and exploratory only. Furthermore, the absence of an in-person control group precludes direct comparison between consultation modalities, and inter- and intra-operator repeatability was not evaluated because consultations were conducted as part of routine care. Second, potential selection biases restrict generalizability. Self-referral via an online platform and the requirement for digital participation likely favored individuals who were already digitally literate and able to engage with telemedicine, potentially resulting in the underrepresentation of those with lower digital proficiency. Additionally, the consultation fee may have created a socioeconomic barrier, potentially limiting access for individuals with fewer financial resources, consistent with previously reported inequalities in access to telemedicine care.^27,28,52^ It also cannot be excluded that paying for the service influenced satisfaction ratings by shaping perceived value and expectations, as pricing has been associated with patient satisfaction and subjective evaluations of benefit.^53^ Moreover, the recommendation that individuals with pre-existing neurological disorders refrain from self-booking a TVC appointment, intended to better isolate symptoms temporally associated with SARS-CoV-2 infection or COVID-19 vaccination, likely introduced further selection bias. This may limit the generalizability of the findings to broader patient populations, in whom neurological and psychiatric comorbidities are common and may interact with, or be exacerbated by, PCC or post-vaccination symptom presentations. At the same time, because this recommendation was not strictly enforced and some patients reported pre-existing neurological conditions such as migraine or disc herniation, residual clinical confounding cannot be excluded. Severe neurological or psychiatric comorbidities nevertheless remained exclusion criteria in accordance with the ethics approval, and one patient meeting these criteria was excluded from the study. Finally, data collection relied on self-reports obtained in a routine care setting, potentially resulting in incomplete records. In addition, the post-consultation questionnaire was developed for internal quality management and was not psychometrically validated. The findings from this questionnaire therefore reflect patient-reported experience rather than objective service quality, clinical suitability, or effectiveness. The study also did not assess objective outcomes such as diagnostic accuracy, safety, follow-up adequacy, or clinical effectiveness, nor did it systematically capture adverse events, missed diagnoses, delayed care, re-presentations, or downstream clinical outcomes. Given the high symptom burden, caregiver assistance was required in several consultations. While often necessary, caregiver involvement may have influenced communication dynamics, privacy, and patient-reported outcomes.^54^

Large-scale, controlled, and longitudinal studies comparing TVC with in-person consultations are needed to assess clinical effectiveness and safety. Further research is required to strengthen remote neurological assessment through validated neurology-specific instruments, complementary remote monitoring approaches, and standardized protocols.^51^ The further development of telemedical care will also depend on user-friendly platforms, standardized procedures, and robust infrastructure. By potentially mitigating barriers such as geographical distance and severe fatigue, TVC may represent a supportive entry point into specialist care for selected patients.^55^ A growing body of literature in clinical neurology describes potential applications of TVC across different clinical contexts.^31,56–58^ Related developments have also been reported in other medical specialties including respiratory medicine, where telemedicine has been associated with support for continuity of care and treatment adherence in selected settings, for example, among patients with CPAP adaptation difficulties.^59^

## 5. Conclusion

These exploratory findings suggest a positive immediate patient-reported experience with a structured TVC pathway among patients presenting with neurological symptom onset in temporal association with either COVID-19 disease or COVID-19 vaccination in a real-world routine neurological care setting. Across the cohort, fatigue and subjective cognitive difficulties were prominent. While this study was not designed to establish clinical effectiveness or equivalence to in-person care, it outlines a structured specialist workflow integrating remote history taking, digital symptom assessment, document-based pre-review, brief cognitive screening, and referral for on-site evaluation where clinically indicated. Within the limits of this study, TVC may represent a supportive entry point for specialist assessment, referral guidance, and care navigation in this selected patient group, while remaining complementary to in-person neurological examination. Interpretation is limited by the small, single-center, highly selected, self-referred, digitally enabled, fee-based cohort and by the use of non-validated patient-experience measures. Further controlled and longitudinal studies are needed to determine safety, clinical utility, and equitable implementation.

## Supplemental material

Supplemental material - Specialist care at a distance: Patient-reported experience with telemedicine video consultations for neurological symptoms associated with post-COVID-19 condition or in temporal association with COVID-19 vaccination in a single-center retrospective cross-sectional study Supplemental material for Specialist care at a distance: Patient-reported experience with telemedicine video consultations for neurological symptoms associated with post-COVID-19 condition or in temporal association with COVID-19 vaccination in a single-center retrospective cross-sectional study by Vanessa Raeder, Fabian Boes, Benno Bremer, Ameli Breuer, Heinrich Audebert and Christiana Franke in Digital Health.

Supplemental material - Specialist care at a distance: Patient-reported experience with telemedicine video consultations for neurological symptoms associated with post-COVID-19 condition or in temporal association with COVID-19 vaccination in a single-center retrospective cross-sectional study Supplemental material for Specialist care at a distance: Patient-reported experience with telemedicine video consultations for neurological symptoms associated with post-COVID-19 condition or in temporal association with COVID-19 vaccination in a single-center retrospective cross-sectional study by Vanessa Raeder, Fabian Boes, Benno Bremer, Ameli Breuer, Heinrich Audebert and Christiana Franke in Digital Health.

## Data Availability

The original contributions presented in the study are included in the article, further inquiries can be directed to the corresponding author.*
