# Supplementary material for: Specialist care at a distance: Patient-reported experience with telemedicine video consultations for neurological symptoms associated with post-COVID-19 condition or in temporal association with COVID-19 vaccination in a single-center retrospective cross-sectional study
Source: Digit Health. 2026 Jun 8;12:20552076261451372. doi: 10.1177/20552076261451372 (PMC13247382; doi:10.1177/20552076261451372)
Supplement: Supplemental material - Specialist care at a distance: Patient-reported experience with telemedicine video consultations for neurological symptoms associated with post-COVID-19 condition or in temporal association with COVID-19 vaccination in a single-center retrospective cross-sectional study [file sj-pdf-1-dhj-10.1177_20552076261451372.pdf]

COVID-19 Vaccination Distribution in patients with neurological symptom onset in temporal association with COVID-19 disease (PwPCC) or COVID-19 vaccination (PwPV): Number and Percentage of Vaccinations and Onset of Symptoms by Vaccine Type

COVID-19 vaccine	PwPCC - vaccination		PwPV - vaccination		PwPV – first onset of symptoms	
	n	%	n	%	n	%
**1. vaccination**						
BNT162b2	22	84.6%	11	73.3%	7	46.7%
mRNA-1273	2	7.7%	2	13.3%	0	0.0%
ChAdOx1-S	1	3.8%	2	13.3%	1	6.7%
N/A	1	3.8%	0	0.0%	0	0.0%
Not Vaccinated	0	0.0%	0	0.0%	0	0.0%
**2. vaccination**						
BNT162b2	23	88.5%	6	40.0%	0	0.0%
mRNA-1273	2	7.7%	3	20.0%	2	13.3%
ChAdOx1-S	0	0.0%	1	6.7%	1	6.7%
N/A	1	3.8%	0	0.0%	0	0.0%
Not Vaccinated	0	0.0%	5	33.3%	0	0.0%
**3. vaccination**						
BNT162b2	11	42.3%	7	46.7%	2	13.3%
mRNA-1273	6	23.1%	0	0.0%	0	0.0%
ChAdOx1-S	0	0.0%	1	6.7%	1	6.7%
N/A	1	3.8%	0	0.0%	0	0.0%
Not Vaccinated	8	30.8%	7	46.7%	0	0.0%
**4. vaccination**						
BNT162b2	3	11.5%	3	20.0%	1	6.7%
mRNA-1273	0	0.0%	0	0.0%	0	0.0%
ChAdOx1-S	0	0.0%	0	0.0%	0	0.0%
N/A	1	3.8%	0	0.0%	0	0.0%
Not Vaccinated	22	84.6%	12	80.0%	0	0.0%
**Combinations**						
BNT162b2	17	65.4%	10	66.7%		
mRNA-1273	0	0.0%	2	13.3%		
BNT162b2, mRNA-1273	7	26.9%	0	0.0%		
ChAdOx1-S, BNT162b2	0	0.0%	2	13.3%		
ChAdOx1-S, BNT162b2, mRNA-1273	1	3.8%	1	6.7%		
N/A	1	3.8%	0	0.0%		

Abbreviations: PwPCC: patients with neurological symptom onset in temporal association with COVID-19 disease; PwPV: patients with neurological symptom onset in temporal association with COVID-19 vaccination.
