# Supplementary material for: Specialist care at a distance: Patient-reported experience with telemedicine video consultations for neurological symptoms associated with post-COVID-19 condition or in temporal association with COVID-19 vaccination in a single-center retrospective cross-sectional study
Source: Digit Health. 2026 Jun 8;12:20552076261451372. doi: 10.1177/20552076261451372 (PMC13247382; doi:10.1177/20552076261451372)
Supplement: Supplemental material - Specialist care at a distance: Patient-reported experience with telemedicine video consultations for neurological symptoms associated with post-COVID-19 condition or in temporal association with COVID-19 vaccination in a single-center retrospective cross-sectional study [file sj-pdf-2-dhj-10.1177_20552076261451372.pdf]

A comparative analysis of the benefits and barriers for patients, caregivers and healthcare professionals in the utilisation of video consultation

Benefits

Patients and Caregivers

- Easy accessibility and self-scheduling appointments
- Attendance from home or bed possible
- Eliminated travel saves time, effort, and costs
- Less stress and higher patient comfort in a familiar environment
- Personalized care tailored to individual needs
- Involvement of family members and caregivers
- Expanded access to specialized clinics globally
- Lower resource consumption (less fuel use, no masks during pandemics)
- Minimized risk of infection
- Reduced pressure by completing questionnaires at home in advance
- Home environment comfort facilitated open communication and detailed medical histories
- Participation in research without geographical constraints
- Improved healthcare delivery over a large area

Healthcare Professionals

- Easy documentation and record keeping
- Ability to view medical reports in advance for better preparation
- Automated evaluation of questionnaires highlighting abnormalities
- Calmer and less stressed patients
- Easy digital data exchange and implementation of additional tools
- Fewer personnel needed
- Ability to see patients unable to visit in person
- Cost-effective and lower resource consumption (less fuel use, no masks during pandemics)
- Minimized risk of infection
- Virtual waiting room enhances efficiency
- Cancellation fees for unattended appointments
- Expanded access as telemedicine extends the catchment area
- Improved healthcare delivery over a large area

Barriers

Patients and Caregivers

- Technical issues and required technical know-how
- Need for equipment and fast internet
- Internet connectivity issues
- Data security and privacy concerns
- Limited physical examination capabilities
- Perception that telemedicine cannot fully replicate in-person consultations
- Potential feelings of isolation for patients with limited social contacts
- Digital dependency problematic for those unfamiliar with technology, especially older individuals
- Financial burden due to private treatment

Healthcare Professionals

- Adapting to new technologies and workflows
- Clinic investment and ongoing platform costs
- Missing or non-digitized documents
- Technical challenges: data protection issues, technology handling, and need for staff training
- Integration into central management systems requires additional resources
- Limited physical examination capabilities due to no physical presence and tactile interaction
- Ethical aspects of treating financially burdened patients
